# Study on a Thermally Crosslinking Clay-Free Weak Gel Water-Based Drilling Fluid

**DOI:** 10.3390/gels12040280

**Published:** 2026-03-27

**Authors:** Taifeng Zhang, Jinsheng Sun, Kaihe Lv, Jingping Liu, Lei Nie, Yufan Zheng, Yuanwei Sun, Ning Huang, Delin Hou, Han Yan, Yecheng Li

**Affiliations:** 1 State Key Laboratory of Deep Oil and Gas, China University of Petroleum (East China), Qingdao 266580, China; 17320011560@163.com (T.Z.);; 2 School of Petroleum Engineering, China University of Petroleum (East China), Qingdao 266580, China

**Keywords:** clay-free water-based drilling fluids, three-dimensional network, weak gel, high temperature, high salinity, thermal crosslinking

## Abstract

In this study, a thermally crosslinking clay-free weak gel water-based drilling fluid based on salt-responsive polymers and crosslinking agents was investigated as a promising and feasible strategy. Firstly, a salt-tolerant polymer was synthesized using N,N-dimethylacrylamide (DMAA), [2-(methacryloyloxy)ethyl]dimethyl-(3-sulfonopropyl)ammonium hydroxide (DMAPS), and acrylamide (AM). BPEI_10,000_ was selected as the thermal crosslinking agent. The optimal crosslinking was achieved at 180 °C and 36% NaCl, with RMFL at 2.0% and BPEI_10,000_ at 0.1%. Performance evaluation demonstrated that the crosslinking between RMFL and BPEI_10,000_ could enhance the AV, PV, and YP of the RMFL(BPEI_10,000_)/CF-WBDFs after aging at 180 °C for 16 h and reduce FL_API_. The RMFL(BPEI_10,000_)/CF-WBDFs exhibited appropriate shear-thinning behavior, viscoelasticity, thixotropy, and recoverable viscosity under high-temperature, high-salinity, and high-pressure conditions. Mechanism analysis revealed that RMFL and BPEI_10,000_ could form a predominantly negatively charged, three-dimensional crosslinking weak gel at high temperatures. The crosslinking weak gel could form dense filter cakes, improving rheological properties and reducing filtration loss of CFWBDFs in high-temperature, high-salinity environments. This paper proposed a novel method to address the technical challenge of rheological performance failure of CFWBDFs, offering valuable insights for subsequent investigations.

## 1. Introduction

Oil and natural gas are essential energy sources on which human societies depend. They will continue to play a key role in the energy landscape for the foreseeable future. As the pace of oil and gas exploration and development accelerates, shallow and medium depth reservoirs are becoming increasingly depleted. Deep and ultra-deep oil and gas account for about 34% of total geological resources worldwide, representing abundant, high-potential resources. Deep and ultra-deep resources are a viable alternative energy source for the future, making the exploration and development of deeper layers an inevitable trend in the oil and gas industry [1]. Drilling is the first process in deep oil and gas development and remains the central engineering in exploration and production.

However, the harsh geological conditions at greater depths posed significant obstacles to drilling engineering. Drilling fluids are the circulating working fluids used in drilling operations, such as balancing formation pressure, maintaining wellbore stability, suspending cuttings, and lubricating drill bits. Safe and efficient deep drilling requires high-performance drilling fluids [2]. Drilling fluids are generally classified into water-based and oil-based systems. Oil-based drilling fluids provide better resistance to high temperatures and high salinity. They are the preferred choice for drilling through harsh formations. However, oil-based drilling fluids have several shortcomings, including cost, environmental pollution, and difficulty in recycling. Drilling engineers are increasingly concerned about water-based drilling fluids due to their lower cost and environmental advantages. Clay-free water-based drilling fluids (CFWBDFs) do not contain clay-based materials. They offer better reservoir protection and faster drilling speeds. CFWBDFs can avoid the negative effects of clay aggregation or dispersion on their performance, particularly under high-temperature and high-salinity conditions. Therefore, CFWBDFs present promising opportunities for research and applications [3]. However, rheological modifiers are prone to failure under high-temperature and high-salinity conditions. The rheological properties of CFWBDFs will deteriorate, leading to serious downhole problems, such as stuck pipe and wellbore collapse. Severe accidents will prevent the safe and efficient production of deep oil and gas.

Rheology modifiers for CFWBDFs are mainly classified into modified natural materials and synthetic polymers. Ali [4] developed a lignin-based biopolymer (BioDrill FM400) that served as a rheology modifier and fluid-loss reducer. The temperature resistance of BioDrill FM400 was 150 °C. When BioDrill FM400 was blended with xanthan gum (XC), it effectively improved PV, YP, and gel strength in CFWBDFs. Li et al. [5] used tara gum as a rheology modifier in solid-free water-based drilling fluids (SFWBDFs), and the SFWBDFs achieved a temperature resistance of 140 °C. Liu [6] prepared a hydrophobic-modified hydroxyethyl cellulose (HMHEC) as a rheology modifier. HMHEC also exhibited superior high-temperature resistance and high-salinity tolerance, with improved rheological control properties relative to PAM and XC, making HMHEC a green, sustainable alternative to traditional rheology modifiers in CFWBDFs. Ali et al. [4] used carboxymethyl cassava starch (CMTS) as a rheology modifier and filtration-loss reducer in CFWBDFs. The high-temperature, high-pressure filtration loss (FL_HTHP(126°C, 500psi)_) was reduced to 11.0 mL at a CMTS concentration of 2.29 wt%. Although modified natural materials are widely available and environmentally friendly, they contain plenty of ether, glycosidic, and ester bonds that easily degrade at higher temperatures (150 °C). Even when sulfonation, grafting, crosslinking, and other chemical modification strategies are employed to enhance the thermal resistance of materials to 180 °C, such improvements often come at the expense of complex processing procedures and high costs. Due to insufficient thermal stability, existing naturally modified materials cannot be applied in deep formations.

Synthetic polymers offer high design flexibility, with adjustable numbers and types of functional groups and molecular weights [7]. Rheology modifiers with high-temperature and high-salinity resistance could be developed by copolymerizing vinyl monomers using hydrophobic, crosslinking, and zwitterionic modifications [8]. Davoodi [9] synthesized a hydrophobic copolymer (SBASC). Under 25.0% NaCl and 5.7% KCl composite salt conditions, CFWBDFs containing 1% SBASC showed higher PV and YP after aging at 121 °C, with FL_API_ and FL_HTHP_ decreasing by 38.8% and 47.5%. Zhang [10] developed a hydrophobic associative polymer (SSZN). SSZN displayed excellent compatibility with XC, white bitumen, and modified starch. The CFWBDFs exhibited a PV of 87.0 mPa·s, YP of 11.5 Pa, and FL_HTHP(150°C, 3.5MPa)_ of 9.7 mL after aging at 150 °C. Wang [11] synthesized a zwitterionic thickener (FPOD), which showed good compatibility in saturated calcium bromide (CaBr_2_) completion fluids. When the concentration of FPOD was 1% in saturated CaBr_2_ completion fluids, the AV was 37.0 mPa·s after aging at 150 °C. Xie et al. [12] synthesized a viscosifier (SDKP) for CFWBDFs. SDKP exhibited temperature resistance up to 160 °C and salt resistance up to 15% NaCl. After aging at 160 °C, the CFWBDFs containing 1% SDKP exhibited an AV of 56.0 mPa·s, PV of 37.0 mPa·s, YP of 19.0 Pa, and FL_HTHP(120°C, 3.5MPa)_ of 12.8 mL. Although existing polymers exhibit good thermal resistance (up to 160 °C) and salt tolerance (up to 15% NaCl), they do not perform effectively at 180 °C and saturated NaCl conditions.

Most polymers are prone to degradation at high temperatures, including scission of main and side chains, which can impair their thickening properties. Especially in high-salinity environments, a large amount of electrolytes compresses the diffuse double layer of polymers, causing the polymer chains to curl and further weakening their rheology-modifying ability. Although slightly crosslinked, hydrophobic and zwitterionic modifications can enhance the thermal and salt resistance of polymers. However, these measures still cannot prevent thermal degradation of polymers at high temperatures. Ultimately, the rheological performance of clay-free water-based drilling fluids deteriorates. Therefore, it is urgent to seek a novel approach to enhance the salt tolerance and high-temperature stability of polymers. Cui improved the temperature resistance of polyacrylamide from 140 °C to 200 °C using the “Crosslinking compensation” method [13]. “Crosslinking compensation” involves crosslinking polymers with crosslinkers at high temperatures to combat polymer hydrolysis and degradation, thereby maintaining the rheological properties of drilling fluids. Therefore, guided by the “crosslinking compensation” method, we can design a salt-tolerant polymer and crosslink it with a crosslinking agent at high temperatures to mitigate polymer degradation, thereby enabling rheological control of clay-free water-based drilling fluids under high-temperature and high-salinity conditions [14]. The shortcomings of existing technologies and the advantages of this study have been summarized in Table 1 and Table 2.

In this study, a thermally crosslinking clay-free, weak gel, water-based drilling fluid was formulated with a salt-resistant rheology modifier, RMFL, and a crosslinking agent, polyethyleneimine (PEI). We preliminarily investigated the effect of thermal crosslinking between RMFL and PEI on the properties of clay-free water-based drilling fluids, providing a new perspective for the design of rheology modifiers, drilling fluid formulation, and performance regulation.

## 2. Results and Discussion

### 2.1. Effect of DMAM and DMAPS on the Salt Resistance of Polymers

Polyacrylamide (PAM) is a thickening agent widely used in oilfield operations, including drilling, profile control, water blocking, oil displacement, and fracturing [15]. However, PAM’s resistance to high temperature and salinity was insufficient. N,N-Dimethylacrylamide (DMAA) contains rigid dimethyl groups, providing better thermal resistance than AM. The steric hindrance from these dimethyl groups increases intermolecular repulsion, preventing polymer chains from curling and from hydrolysis in high-temperature, high-salinity environments. DMAA is more suitable than AM as a backbone for high-temperature- and high-salinity-resistant polymers [16,17,18]. Zwitterionic polymers are polyelectrolytes that contain both cationic and anionic groups. In electrolyte solutions, electrostatic repulsion between these groups is shielded, allowing polymer chains to fully extend. This results in increased drilling fluid viscosity and shear strength, thereby enhancing rheological properties [19]. The effects of PAM, PDMAA, and PDMASs on solution properties were evaluated at different NaCl concentrations, with a polymer concentration of 3.0 wt%.

**Figure 1:**
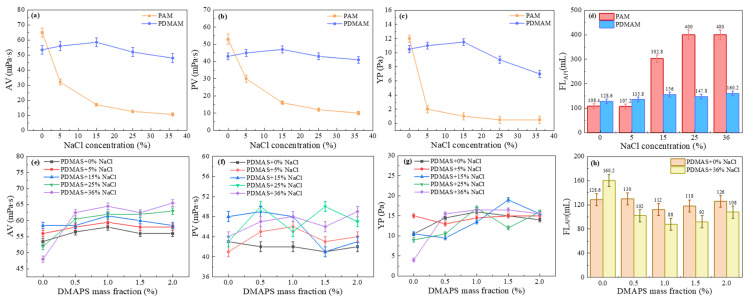
Influence of DMAA and DMAPS on the salt resistance of polymers. (**a**–**d**) Comparison of the viscosity-enhancing and filtration-loss reduction abilities of PDMAM and PAM at different NaCl concentrations; (**e**–**h**) effect of DMAPS mass fraction on the viscosity enhancement and filtration-loss reduction of PDMAS at different NaCl concentrations.

As shown in Figure 1a–c, the AV, PV, and YP of the PAM/CFWBDFs reduced significantly with increasing NaCl concentration from 0% to 36%. As shown in Figure 1d, the FL_API_ of the PAM solution increased with increasing NaCl concentration, with all filtration losses observed at 25% NaCl. Under positive pressure, the polymers could form filter cakes that seal micropores and microcracks. A higher polymer molecular weight or a larger hydrodynamic volume enhanced the sealing effect on micro/nanopores within the filter cake, resulting in a denser cake and reduced filtration loss [20]. Greater molecular weight also strengthened the binding effect of highly hydrating groups (such as amide and sulfonate groups) on free water, further reducing the filtration loss. PAM has good viscosity-increasing properties in freshwater. However, a higher concentration of Na^+^ caused PAM chains to curl, weakening the viscosity enhancement and the filtration-loss reduction properties. Surprisingly, there were no significant declines in AV, PV, or YP of PDMAA/CFWBDFs with increasing NaCl concentration, and higher NaCl concentrations did not increase FL_API_. It was indicated that PDMAA exhibited better salt tolerance than PAM. From Figure 1e–h, it was clear that the AV of PDMAS/CFWBDFs increased as the NaCl concentration rose after adding the DMAPS to the PDMAA molecular chains. The PV stayed within a narrow range of 40.0 to 48.0 mPa·s, and the YP ranged from 9.5 to 16.5 Pa. When the mass fraction of DMAPS was 1.0 wt%, PDMAS exhibited the best performance compared to PDMAA.

**Figure 2:**
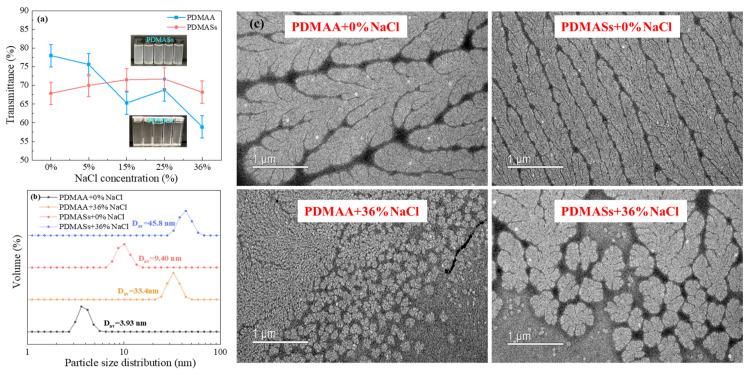
Microscopic mechanism analysis of the salt tolerance of PDMAA and PDMASs. (**a**) Solution transmittance analysis; (**b**) polymer particle size analysis; (**c**) polymer micromorphology analysis.

As shown in Figure 2a, the transmittance of the PDMAA/CFWBDFs decreased steadily with increasing NaCl concentration. The transmittance of PDMAS/CFWBDFs remained mainly stable at around 70%. The PDMAM/CFWBDFs became slightly turbid as NaCl concentration increased, while the PDMAS/CFWBDFs shifted from clear to slightly turbid. This phenomenon may be due to the formation of hydrophobic regions by PDMAM and PDMAS in solution, thereby decreasing light transmittance [21].

From Figure 2b, it was shown that, at 0% NaCl, the average particle sizes (D_av_) of PDMAA and PDMAS were 3.93 nm and 9.40 nm, respectively. At 36% NaCl, D_av_ increased to 33.4 nm for PDMAA and 45.8 nm for PDMAS. It was suggested that NaCl promoted polymer extension, allowing polymers to adsorb more water, and the D_av_ of polymers was enlarged.

As shown in Figure 2c, both PDMAA and PDMAS had slender, willow-like shapes at 0% NaCl. More side chains were stretching out; the polymers were fully extended in a high-salinity environment. After the polymer chains extended, they enhanced the interpolymer interactions, thereby forming a three-dimensional associative network. The network likely resulted from weak hydrophobic interactions of dimethyl groups in DMAA. A higher concentration of Na^+^ increased solution polarity and promoted hydrophobic association among a small number of dimethyl groups, forming a three-dimensional associative network [22]. Undoubtedly, PDMAA/CFWBDFs exhibited higher AV, PV, and YP and lower FL_API_ than PAM/CFWBDFs under high-salinity conditions. In addition, PDMASs possessed betaine side chains, which extended and led to a larger hydrodynamic radius in a high-salinity environment [23], thereby enhancing AV, PV, and YP and reducing filtration loss.

### 2.2. Effect of AM Mass Fraction on the Properties of the Crosslinked System

Polyethyleneimine was introduced to crosslink polymers via transamidation at high temperatures, thereby preventing polymer degradation and performance failures due to high-temperature hydrolysis. Polyethyleneimine (PEI) is an aziridine polymer containing abundant imine groups. These imine groups are highly reactive and easily crosslink with functional groups in polymers, such as amide, carboxyl, or hydroxyl groups. PEI is often used as the crosslinker in oilfield operations, including profile adjustment, water blocking, and fracturing [24]. The number of amide groups, which serve as crosslinking sites, will influence the crosslinking efficiency between the polymer and PEI. Under conditions of 180 °C and 36% NaCl, the crosslinked product between synthesized polymers (PDMASA-0, PDMASA-1, PDMASA-2, PDMASA-3, and PDMASA-4) and 0.25 wt% (*w*/*v*) LPEI_1800_ after aging was observed. The rheological and filtrate loss properties of each sample were evaluated.

**Figure 3:**
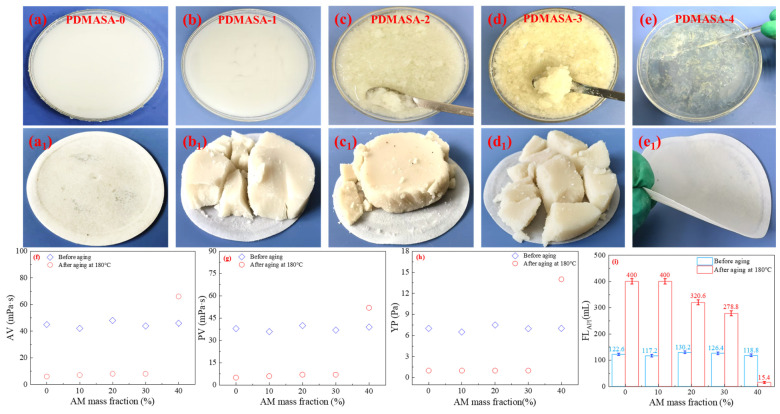
Effect of AM mass fractions on the properties of the crosslinked system under 36% NaCl and 180 °C conditions. (**a**–**e**) The appearance of the polymers and the LPEI_1800_ crosslinked system after aging; (**a_1_**–**e_1_**) appearance of the filter cakes of crosslinked systems; (**f**–**i**) AV, PV, and YP of polymer(LPEI_1800_)/CFWBDFs.

From Figure 3a–e, under 36% NaCl conditions, PDMASA-0(LPEI_1800_)/CFWBDFs and PDMASA-1(LPEI_1800_)/CFWBDFs appeared milky white and highly diluted after aging at 180 °C. In contrast, many macroscopic gel particles existed in the PDMASA-2(LPEI_1800_)/CFWBDFs and PDMASA-3(LPEI_1800_)/CFWBDFs. Surprisingly, the PDMASA-4(LPEI_1800_)/CFWBDFs formed a gel that did not flow after aging [25]. As demonstrated in Figure 3f–h, under 180 °C and 36% NaCl conditions, the combining systems of PDMASA-0, PDMASA-1, PDMASA-2, and PDMASA-3 with 0.25 wt% LPEI_1800_ exhibited an AV of 6.0 to 8.0 mPa·s, a PV of only 5.0 to 7.0 mPa·s, and a YP of only 1.0 Pa. The weak gel formed by PDMASA-4 and LPEI_1800_ became a flowable viscous solution after shearing, with an AV of 66.0 mPa·s, a PV of 52.0 mPa·s, and a YP of 14.0 Pa. The results suggested excessive crosslinking between PDMASA-4 and LPEI_1800_.

From Figure 3i, it is clear that the FL_API_ of the systems in Figure 3a–d was 350.0, 350.0, 320.6, and 278.8 mL, respectively. However, the FL_API_ of the system shown in Figure 3e was only 15.4 mL.

The filter cakes from each system are shown in Figure 3(a_1_). The PDMASA-0(LPEI_1800_)/CFWBDFs had a thinner filter cake but experienced significant filtration loss. It was suggested that PDMASA-0, lacking amide groups, could not crosslink with LPEI_1800_ at high temperatures. The filter cakes of the CFWBDFs shown in Figure 3(b_1_–d_1_) were thicker, with a large amount of filtration loss, indicating that the water-insoluble gel particles were formed by PDMASA-1, PDMASA-2, and PDMASA-3 crosslinking with LPEI_1800_. It confirmed that PDMASA-1, PDMASA-2, and PDMASA-3 could crosslink with LPEI_1800_. However, the gel particles could not effectively improve the rheological properties or reduce the filtration loss.

After PDMASA-4 crosslinking with LPEI_1800_, a viscous solution was obtained. It showed increased AV, PV, and YP and a decreased FL_API_. As shown in Figure 3(e_1_), the filter cake was thin, with no visible gel particles on its surface [26]. The results indicated that adding 0.25% LPEI_1800_ (*m*/*v*) to PDMASA polymerization, with an AM mass fraction of 40 wt%, enabled PDMASA and LPEI_1800_ to crosslink [27]. Even with excessive crosslinking, it still aligned with our predicted model. Therefore, the optimal mass fraction for AM polymerization was 40 wt%.

### 2.3. Preparation and Characterization of RMFL

**Figure 4:**
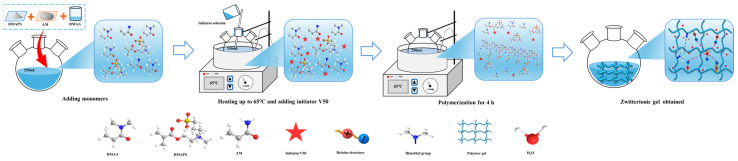
Synthesis of RMFL.

**Figure 5:**
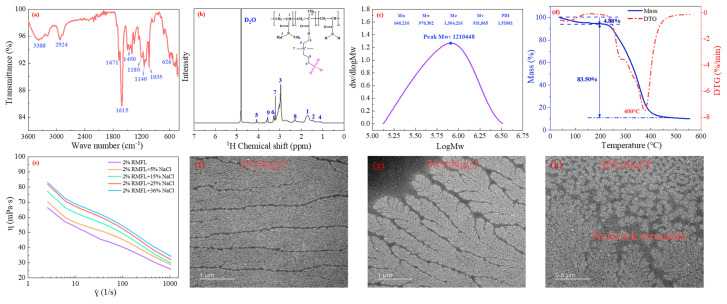
Characterization of RMFL. (**a**) FTIR; (**b**) 1H NMR; (**c**) molecular weight of RMFL; (**d**) TGA analysis; (**e**) rheological curves of RMFL/CFWBDFs with different concentrations of NaCl. (**f**–**h**) Microtopography of RMFL.

The preparation method is shown in Figure 4. The synthesis route was illustrated in the above section. The characterization of RMFL is shown in Figure 5. The infrared spectrum is shown in Figure 5a. The characteristic peak at 3388 cm^−1^ was from N-H stretching in the amide groups; the peaks at 2924 and 2870 cm^−1^ were from C-H stretching in methyl and methylene groups [28]; the peaks at 1671 cm^−1^ were from the C=O stretching in the amide groups; the peak at 1615 cm^−1^ was from N-H bending vibration; the peak at 1450 cm^−1^ was from the N^+^-C stretch in the quaternary ammonium cation; the peaks at 1180 cm^−1^ and 1037 cm^−1^ were from S=O and S-O stretching in the sulfonic acid group; the peak at 628 cm^−1^ was from C-S stretching [29]. Infrared spectroscopy analysis indicated that the product contained amide, methyl, carbonyl, quaternary ammonium, and sulfonic acid groups.

The ^1^H NMR spectrum is shown in Figure 5b; the peak at 1.11 ppm was attributed to the chemical shift of H on the methyl groups attached to the polymer main chains; the peak at 1.53 ppm was from the chemical shift of H in the methylene groups of the main chains; multiple peaks between 1.60 and 1.80 ppm originated from the chemical shift of H on the methylene groups within the main chain [30]; the peak at 2.27 ppm came from the chemical shift of H on the intermediate methylene group of N^+^-CH_2_-CH_2_-CH_2_-SO_3_ [31]; the peaks from 2.80 to 3.20 ppm correspond to the chemical shift of H on -CON(CH_3_)_2_; the peak at 3.20 ppm was related to the chemical shift of H in N^+^(CH_3_)_2_; the peak at 3.28 ppm corresponded to the chemical shift of H in N^+^-CH_2_; the peak at 3.56 ppm resulted from the chemical shift of H in the methylene bridge connected to the sulfonate group within N^+^-CH_2_-CH_2_-CH_2_-SO_3_; the peak at 4.08 ppm could correspond to the H chemical shift of -COO-CH_2_- [32]; ^1^H NMR and FTIR indicated that the product contained functional groups of the monomers. The molecular structure aligned with the design, confirming the successful polymerization of RMFL.

As shown in Figure 5c, the number-average molecular weight (M_n_) of RMFL was 897,940 Da, and the weight-average molecular weight (M_w_) was 1,176,302 Da. The polydispersity index (PDI) was 1.31081, indicating a narrow molecular weight distribution and good polymerization efficiency, which helped to minimize experimental errors. The thermogravimetric (TGA) curve of RMFL is shown in Figure 5d. As the temperature increased from 50 °C to 550 °C, the weight loss of RMFL was mainly divided into three stages. The first stage was from 50 to 197 °C, resulting in approximately 4.88% mass reduction. The mass loss was primarily due to the evaporation of bound water adsorbed on amide and sulfonic acid groups as well as free water between the polymer chains. The second stage was from 197 to 475 °C; approximately 83.50% mass reduction was observed, primarily due to the thermal degradation of amide, sulfonic acid, the quaternary ammonium cation, and methyl groups. The third stage was from 475 °C to 550 °C; RMFL exhibited less mass loss, with a decrease of 1.87%. During this stage, the backbone of RMFL underwent scission until the polymer was completely carbonized.

The microtopography of RMFL is shown in Figure 5f–h. From Figure 5f, RMFL displayed a fine and willow-branch-like structure under 0% NaCl conditions, which can be attributed to the electrostatic attraction between the anionic and cationic groups on the polymer side chains. The electrostatic attraction led to the contraction of the side chains. In Figure 5g, the side chains of RMFL become extended under 36% NaCl conditions because the electrostatic attraction between quaternary ammonium cations and sulfonate groups was shielded by a large amount of Na^+^. Furthermore, under saturated salt conditions, the network structure formed by RMFL was observed, as shown in Figure 5h, which may result from intermolecular electrostatic and hydrophobic interactions among RMFL molecules, with electrostatic interactions being dominant. Such a network structure contributes to the regulation of the rheological properties of clay-free water-based drilling fluids.

### 2.4. Effect of Polyethyleneimine on the Performance of RMFL

**Figure 6:**
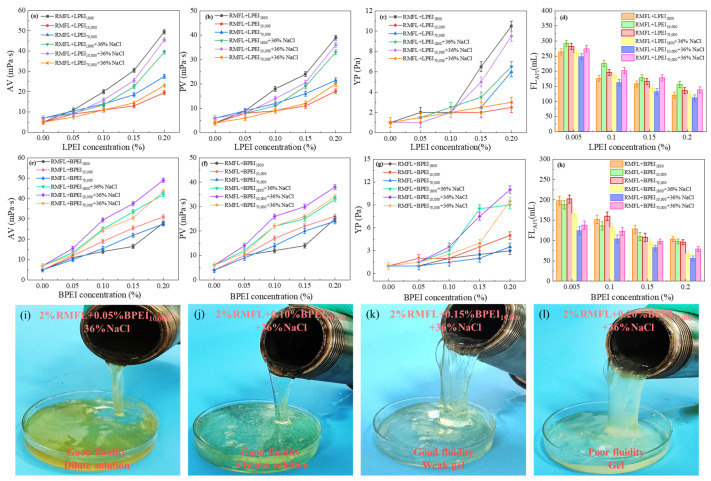
Effect of polyethyleneimine on the performance of RMFL. (**a**–**d**) Effect of LPEI_x_ on the performance of RMFL(LPEI_x_)/CFWBDFs; (**e**–**h**) effect of BPEI_y_ on the performance of RMFL(BPEI_y_)/CFWBDFs; (**i**–**l**) state of RMFL(BPEI_10,000_)/CFWBDFs with different concentrations of BPEI_10,000_.

The performance of the RMFL crosslinked with PEI of different molecular weights, configurations, and concentrations (*w*/*V*) is illustrated in Figure 6. As shown in Figure 6a–d, increasing LPEI_x_ concentration gradually raised the AV, PV, and YP of the RMFL(LPEI_x_)/CFWBDFs after aging at 180 °C for 16 h. The FLAPI gradually decreased. Higher LPEI_x_ concentrations led to increased crosslinking between RMFL and LPEI_x_, enhancing the rheological properties of the RMFL(LPEI_x_)/CFWBDFs and reducing filtration loss.

LPEI_x_ with different molecular weights exhibited different crosslinking effects at the same concentration. At 0% NaCl, LPEI_1800_ demonstrated optimal crosslinking with RMFL, as the RMFL(LPEI_1800_)/CFWBDFs achieved the highest AV, PV, and YP after aging at 180 °C, along with the lowest FL_API_. This difference arises because the three LPEI_x_ have different molarity at the same concentration. The molarity of LPEI_1800_ was 2.22 × 10^−3^ mol/L, the molarity of LPEI_10,000_ was 0.4 × 10^−3^ mol/L, whereas the molarity of LPEI_70,000_ was only 0.057 × 10^−3^ mol/L. Although LPEI_1800_ has a lower molecular weight, its molar concentration is more than five times that of LPEI_10,000_ and thirty-nine times that of LPEI_70,000_. This indicated that the molarity of the crosslinker is the primary factor influencing the crosslinking efficiency in freshwater environments, and it has a greater impact than molecular weight.

However, LPEI_10,000_ was the most effective in crosslinking with RMFL under 36% NaCl conditions. This is because the longer polymer chains of LPEI_10,000_ can simultaneously interact with multiple RMFL chains, facilitating binding and the formation of a more extensive three-dimensional network. In high-salinity environments, electrostatic interactions are strongly screened, so the crosslinking efficiency is mainly determined by the chain length and spatial bridging ability. Therefore, although LPEI_1800_ has a higher molar concentration, its shorter chain length limits the number of effective crosslinking sites. As shown in Figure 6e–h, with increasing BPEI_y_ concentration, the AV, PV, and YP of the RMFL(BPEI_y_)/CFWBDFs gradually increased after aging at 180 °C. The RMFL(BPEI_y_)/CFWBDFs exhibited higher AV, PV, and YP, with the lowest FLAPI. BPEI_y_ demonstrated a superior crosslinking effect compared to LPEI_x_. The results were attributed to the branched structure of BPEI_y_. BPEI_y_ exposed a greater number of highly reactive imine groups at the terminal ends of side chains, thereby increasing the probability of reaction with RMFL. Therefore, BPEI_y_ was more effective than LPEI_x_ in crosslinking with RMFL [33], significantly improving the rheological properties of RMFL(BPEI_y_)/CFWBDFs under high-temperature and high-salinity conditions.

Comparing the effects of BPEI_y_ with different molecular weights on the RMFL(BPEI_y_)/CFWBDFs, BPEI_10,000_ exhibited the most effective crosslinking with RMFL under both 0% NaCl and 36% NaCl conditions. The RMFL(BPEI_10,000_)/CFWBDFs showed the highest AV, PV, and YP and the lowest FL_API_ after aging. This is the combined effect of molecular configuration, molecular weight, and molarity. The crosslinking efficiency of BPEI_70,000_ was lower because of its lower molarity at the same concentration. When the molar concentrations were comparable, such as between BPEI_1800_ and BPEI_10,000_, a higher molecular weight may lead to improved crosslinking performance. Under saturated NaCl conditions, BPEI_1800_ and BPEI_10,000_ were subject to strong charge screening. However, due to its higher molecular weight and larger hydrodynamic volume, BPEI_10,000_ exhibited stronger bridging ability and could simultaneously interact with multiple RMFL chains. In contrast, the smaller hydrodynamic volume of BPEI_1800_ limited it to localized, single-point interactions.

As shown in Figure 6i–l. When the BPEI_10,000_ concentration was 0.05%, the RMFL(BPEI_10,000_)/CFWBDFs formed a dilute solution with good fluidity after aging at 180 °C. At a BPEI_10,000_ concentration of 0.10%, the RMFL(BPEI_10,000_)/CFWBDFs became thicker after aging, exhibiting wall clinging and easy flow, and possessed weak gel-like properties.

At a BPEI_10,000_ concentration of 0.15%, RMFL and BPEI_10,000_ created a gel with a stronger internal network. A “spitting tongue” phenomenon demonstrated significant flow resistance during pouring [34]. At a BPEI_10,000_ concentration of 0.20%, the internal network of the crosslinked polymer became even stronger, forming a cohesive gel. The results indicated that, at BPEI_10,000_ concentrations of 0.10% or higher, the crosslinked polymer network was sufficiently robust to form a solid gel. It was always better to ensure adequate fluid flow during drilling rather than allow solid gel formation. Solid gel formation can cause serious problems, such as drill bit embedding, difficulty pumping fluids, and inefficient rock penetration. In summary, the optimal BPEI_10,000_ concentration was 0.10%. When 0.10% (*m*/*V*) BPEI_10,000_ was crosslinked with 2% RMFL, it did not form a soft gel. Still, it effectively increased the AV, PV, and YP of CFWBDFs under 180 °C and 36% NaCl conditions.

### 2.5. Rheological Properties Evaluation of RMFL(BPEI_10,000_)/CFWBDFs

**Figure 7:**
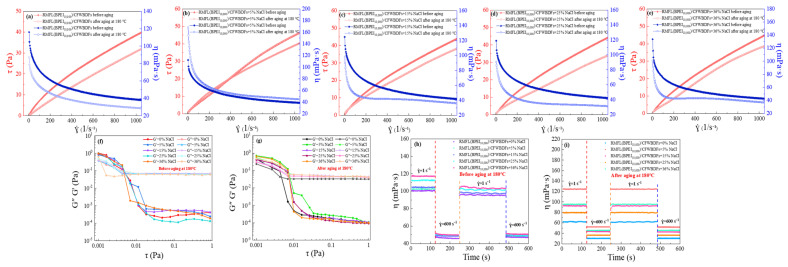
Evaluation of rheological properties of RMFL(BPEI_10,000_)/CFWBDFs. (**a**–**e**) Rheograms of the RMFL(BPEI_10,000_)/CFWBDFs with different concentrations of NaCl before and after aging at 180 °C. (**f**,**g**) Viscoelasticity analysis; (**h**,**i**) thixotropy analysis.

The rheological curves of the RMFL(BPEI_10,000_)/CFWBDFs before and after aging at 180 °C under different NaCl conditions are shown in Figure 7a–e. With increasing NaCl concentration, the viscosity of the RMFL(BPEI_10,000_)/CFWBDFs gradually increased before aging, confirming the anti-polyelectrolyte effect of RMFL. After aging at 180 °C, the viscosity of the RMFL(BPEI_10,000_)/CFWBDFs showed an initial increase, followed by a decrease and then a renewed increase. Under 5% NaCl conditions, the viscosity of the RMFL(BPEI_10,000_)/CFWBDFs reached its highest within the shear rate range of 0–1050 s^−1^. As the NaCl concentration increased to 36%, the viscosity of the RMFL(BPEI_10,000_)/CFWBDFs did not decrease significantly after aging at 180 °C. The results indicated that the RMFL(BPEI_10,000_)/CFWBDFs exhibited temperature resistance and salt tolerance.

The viscoelastic properties of the RMFL(BPEI_10,000_)/CFWBDFs are shown in Figure 7f,g. Within the shear stress range of 0.001 to 0.006 Pa, G′ consistently exceeded G″, indicating the RMFL(BPEI_10,000_)/CFWBDFs mainly exhibited elastic properties. When shear stress exceeded 0.07 Pa, G″ surpassed G′, revealing primarily viscous properties. The results indicated that the RMFL(BPEI_10,000_)/CFWBDFs was a kind of viscoelastic fluid with weak gel properties. Intermolecular forces, such as electrostatic forces and hydrogen bonds between crosslinking polymers, form a weak gel network in the drilling fluid. The weak gel network can resist external shear forces at low shear stress, thereby effectively suspending rock cuttings. The weak gel network could be disrupted as shear stress increased, leading to decreased viscosity and an advantage in breaking rocks. In drilling engineering, the viscoelastic and weak gel properties of RMFL(BPEI_10,000_)/CFWBDFs suspend bit cutting and facilitate bottomhole cleaning [35].

The thixotropy of the RMFL(BPEI_10,000_)/CFWBDFs is shown in Figure 7h,i. At a NaCl concentration of 36%, the viscosity of the RMFL(BPEI_10,000_)/CFWBDFs was 117.07 mPa·s. At the shearing rate of 600 s^−1^, the viscosity decreased to 51.48 mPa·s. Under high shear forces, the three-dimensional weak gel network was disrupted. When the shear rate was restored to 1 s^−1^, the viscosity was 103.33 mPa·s, with a viscosity recovery rate of 88.26%. At low shear rates, the weak gel network in the drilling fluids gradually recovered, driven by van der Waals forces, hydrogen bonds, and electrostatic interactions between crosslinking polymers. After aging, the RMFL(BPEI_10,000_)/CFWBDFs with 36% NaCl exhibited a viscosity of 95.22 mPa·s at 1 s^−1^. At the shear rate of 600 s^−1^, the viscosity was reduced to 45.41 mPa·s. After the shearing rate was restored to 1 s^−1^, the viscosity recovered to 94.34 mPa·s, representing a recovery rate of 99.07%. The RMFL(BPEI_10,000_)/CFWBDFs exhibited thixotropy under high-temperature and high-salinity conditions. The internal weak gel network was disrupted at high shearing rates, reducing viscosity and enabling drilling fluids to be ejected from the drill bit to break rocks. The internal weak gel network rapidly recovered at low shearing rates, allowing drilling fluids to suspend and transport bit cuttings [36].

The Herschel–Bulkley model was used to fit the rheological curves of RMFL(BPEI_10,000_)/CFWBDFs at different NaCl concentrations. The results are presented in Table 3. $\tau_{y}$ represented the yield point of RMFL(BPEI_10,000_)/CFWBDFs. $\tau_{y}$ was 2.089, 2.798, 2.961, 2.738, and 2.596 Pa before aging and was 1.552, 3.043, 2.451, 1.902, and 1.246 Pa after aging at 180 °C. $\tau_{y}$ indicated that the RMFL(BPEI_10,000_)/CFWBDFs possessed a network structure formed by RMFL internally. When the shear stress increased to the yield point, RMFL(BPEI_10,000_)/CFWBDFs were forced to flow. RMFL(BPEI_10,000_)/CFWBDFs exhibited pseudoplastic behavior, with yield points and shear-thinning behavior. RMFL(BPEI_10,000_)/CFWBDFs were capable of breaking rocks at high shear rates and suspending bit cuts at low shear rates.

The results of rheological properties evaluation demonstrated that RMFL(BPEI_10,000_)/CFWBDFs possessed suitable viscoelasticity, thixotropy, and shear-thinning behavior and appropriate AV, PV, and YP for RMFL(BPEI_10,000_)/CFWBDFs, ensuring safe and efficient deep well drilling operations.

**Figure 8:**
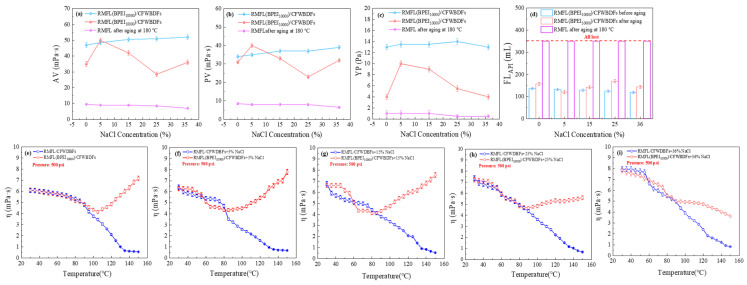
Rheological properties of RMFL(BPEI_10,000_)/CFWBDFs with different concentrations of NaCl before and after aging at 180 °C. (**a**–**d**) AV, PV, YP and FL_API_ of RMFL(BPEI_10,000_)/CFWBDFs. (**e**–**i**) The viscosity–temperature curves for the RMFL(BPEI_10,000_)/CFWBDFs from 30 °C to 150 °C at 500 psi.

Figure 8a–d illustrate the rheological properties and filtration loss of the RMFL(BPEI_10,000_)/CFWBDFs under different NaCl concentrations. As the NaCl concentration increased from 0% to 36%, the PV of the RMFL(BPEI_10,000_)/CFWBDFs was stable at 37.0 mPa·s before aging, and the PV was 31.0, 40.0, 33.0, 23.0, and 32.0 mPa·s after aging at 180 °C, with PV retention rates exceeding 60%. In contrast, the RMFL/CFWBDFs without BPEI_10,000_ exhibited a PV less than 10.0 mPa·s. The YP of the RMFL(BPEI_10,000_)/CFWBDFs was approximately 13.5 Pa before aging. The YP exceeded 4.0 Pa after aging. Without BPEI_10,000_, the YP of the RMFL/CFWBDFs was only 1.0 Pa. In terms of filtration performance, when the NaCl concentration was 5%, 15%, 25%, and 36%, the FL_API_ of RMFL (BPEI_10,000_)/CFWBDFs after aging was 156.6, 122.2, 143.0, 170.8, and 144.6 mL. In contrast, in the absence of BPEI_10,000_, the RMFL/CFWBDFs exhibited complete fluid loss during the API filtration test. The results indicated that BPEI_10,000_ and RMFL effectively modified the rheological behavior and filtration performance of RMFL (BPEI_10,000_)/CFWBDFs. Bridging agents (such as nano-silica and nano-alumina) were not used in this study, resulting in high FL_API_ (>100 mL) of RMFL (BPEI_10,000_)/CFWBDFs. However, nanoparticles could be incorporated into RMFL (BPEI_10,000_)/CFWBDFs to control fluid loss to meet field application standards.

The viscosity–temperature curves of RMFL(BPEI_10,000_)/CFWBDFs are shown in Figure 8e–i. The viscosity of the RMFL/CFWBDFs decreased significantly with temperature increasing from 30 °C to 150 °C. RMFL degraded severely and could not maintain the viscosity of CFWBDFs in the absence of BPEI_10,000_. After adding BPEI_10,000_, the viscosity of the RMFL(BPEI_10,000_)/CFWBDFs first decreased and then increased within the NaCl concentration range of 0% to 25%, with transition temperature points at 115, 118, 128, and 132 °C, respectively. Under 36% conditions, no transition temperature was observed. The viscosity of the RMFL(BPEI_10,000_)/CFWBDFs gradually decreased. However, due to the crosslinking interaction between BPEI_10,000_ and RMFL, the viscosity of the RMFL(BPEI_10,000_)/CFWBDFs remained stable at 3.63 mPa·s at 150 °C and 500 psi, which was increased compared to the RMFL/CFWBDFs without BPEI_10,000_. As NaCl concentration increased, the crosslinking temperature of BPEI_10,000_ with RMFL shifted towards higher temperatures. It could result in the charge-shielding effect of Na^+^ on both BPEI_10,000_ and RMFL, inhibiting the crosslinking reaction between BPEI_10,000_ and RMFL.

The crosslinked polymers of RMFL and BPEI_10,000_ were influenced by the properties of RMFL, BPEI_10,000_, and the salt concentration. In aqueous solution, BPEI_10,000_ ionized to form positively charged amine cations that were minimally shielded by electrolytes. However, the crosslinked polymers were more significantly affected by electrolytes. At 5% NaCl, the crosslinking between RMFL and BPEI_10,000_ was optimal, and the crosslinked polymer exhibited the greatest viscosity-increasing effect. As the NaCl concentration increased, the viscosity of RMFL(BPEI_10,000_)/CFWBDFs decreased. High concentrations of Na^+^ caused charge screening in the crosslinked polymers, leading to curling. However, due to the anti-polyelectrolyte effect, RMFL gradually extended as the NaCl concentration increased. When the NaCl concentration reached 36%, the RMFL was fully extended. Although the charges on the RMFL molecular segments are shielded, the fully extended RMFL retained effective crosslinking capability with BPEI_10,000_, thereby improving the rheological properties of the RMFL(BPEI_10,000_)/CFWBDFs under high-temperature, high-salinity conditions.

### 2.6. Evaluation of RMFL(BPEI_10,000_)/CFWBDFs Under High-Density Conditions

**Figure 9:**
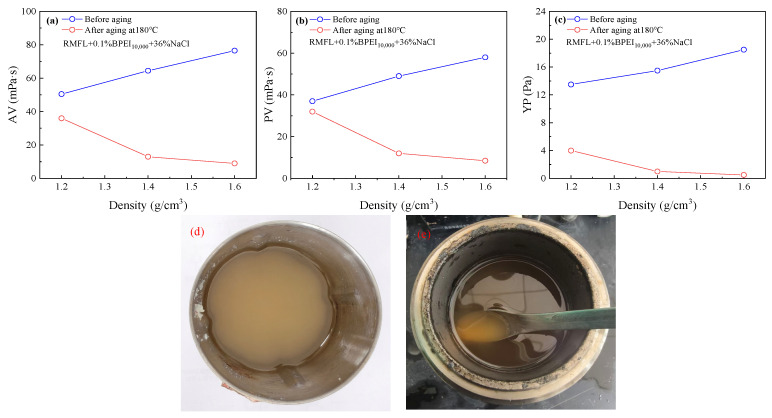
Influence of density on the performance of RMFL (BPEI_10,000_)/CFWBDFs. (**a**–**c**) Influence of density on the rheological properties. (**d**,**e**) Appearance of RMFL (BPEI_10,000_)/CFWBDFs after aging at 180 °C.

The results are shown in Figure 9. As illustrated in Figure 9a–c, the AV, PV, and YP of the drilling fluid gradually decreased with increasing density. When the drilling fluid density was 1.40 g/cm^3^, the PV dropped sharply to 13.0 mPa·s after aging, and the YP was only 1.0 Pa. At a density of 1.60 g/cm^3^, the PV further decreased to 9.0 mPa·s after aging, with the YP reduced to only 0.5 Pa. From Figure 9d,e, barite settled significantly after aging, indicating that the internal network structure of RMFL(BPEI_10,000_)/CFWBDFs failed.

It was suggested that the crosslinking efficiency between RMFL and BPEI_10,000_ was weakened with increasing drilling fluid density, thereby diminishing the rheological enhancement effect of the crosslinking polymer. The phenomenon was mainly due to barite, a large inert solid particle, which created pronounced steric hindrance within the drilling fluids, interfering with effective crosslinking between RMFL and BPEI_10,000_.

**Figure 10:**
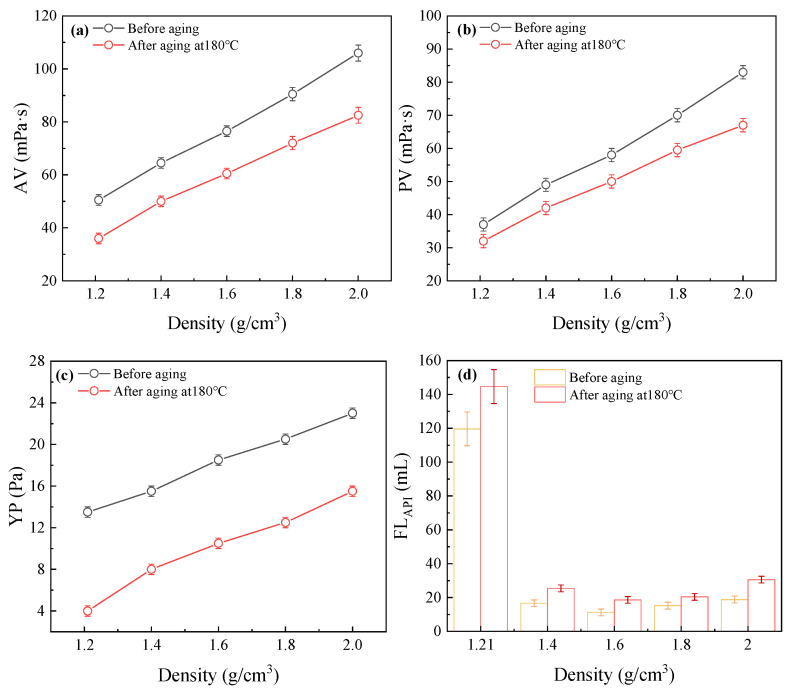
Performance evaluation of different density RMFL(BEPI_10,000_)/CFWBDFs. (**a**) AV; (**b**) PV; (**c**) YP; (**d**) FL_API_.

As discussed previously, increasing the molar concentration of BPEI_10,000_ can enhance the crosslinking efficiency between RMFL and BPEI_10,000_. Therefore, the RMFL/BPEI_10,000_ ratio was optimized for high-density drilling fluids, and an appropriate ratio suitable for high-density barite-weighted systems was identified. As shown in Figure 10, the recommended mass ratio of RMFL to BPEI_10,000_ was 1:0.10, for RMFL(BPEI_10,000_)/CFWBDFs with a density of 1.40 g/cm^3^. When the density was increased to 1.60 g/cm^3^, the recommended mass ratio of RMFL to BPEI_10,000_ was 1:0.15. For RMFL (BPEI_10,000_)/CFWBDFs with densities of 1.80 and 2.00 g/cm^3^, the recommended mass ratios of RMFL to BPEI_10,000_ were 1:0.25 and 1:0.50. After optimization of the RMFL/BPEI_10,000_ ratio, the basic properties of RMFL (BPEI_10,000_)/CFWBDFs under saturated salt conditions before and after aging at 180 °C. It could be observed that RMFL (BPEI_10,000_)/CFWBDFs maintained good rheological properties after aging. In addition, the presence of barite significantly reduced the filtration loss.

**Figure 11:**
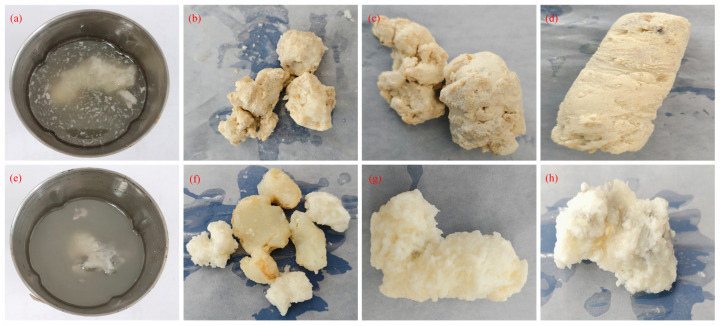
Compatibility of the RMFL(BPEI_10,000_)/CFWBDFs with Ca^2+^ and Mg^2+^. (**a**–**d**) Compatibility of BPEI_10,000_ with different concentrations of calcium chloride; (**e**–**h**) compatibility of branched polyethyleneimine with different concentrations of calcium bromide.

Further investigations were conducted to evaluate the performance of RMFL and BPEI_10,000_ in Ca^2^-based CFWBDFs. The concentrations of RMFL and BPEI_10,000_ were fixed at 2.0% and 0.1%, as shown in Figure 11. Figure 11a shows the appearance of the CaCl_2_ drilling fluid with a density of 1.26 g/cm^3^ after aging at 180 °C. As shown in Figure 11b–d, bulk insoluble aggregates were observed. Large insoluble precipitates were observed in all CaCl_2_ drilling fluids with densities of 1.35, 1.41, and 1.43 g/cm^3^. As shown in Figure 11e–h, the same phenomenon was also observed in CaBr_2_ drilling fluids of different densities. With increasing amounts of CaCl_2_ and CaBr_2_, more precipitates formed, and the viscosity of CFBWDFs decreased to a nearly negligible level. This was because BPEI_10,000_ not only formed a crosslinked polymer network with RMFL but also chelated Ca^2+^. The crosslinked polymer could adsorb a significant amount of Ca^2+^. The abundant Ca^2+^ could strongly chelate with other crosslinked polymers, leading to extensive polymer adsorption. A large, insoluble, complex precipitate eventually formed.

The results indicated that RMFL(BEPI_10,000_)/CFWBDFs were not applicable to environments with high concentrations of Ca^2+^. Their application was currently impractical, though it was mechanistically feasible.

The unsuitability of the RMFL/BPEI_10,000_ system in Ca^2+^-based CFWBDFs indicated that the crosslinker should possess specific crosslinking functionality. It should selectively target and react with the intended polymer to form a crosslinked network rather than reacting with other components in the system. Otherwise, the interactions with other components would undermine the effectiveness of both the polymer and the crosslinker, leading to failure of rheological regulation.

### 2.7. Mechanism Exploration

**Figure 12:**
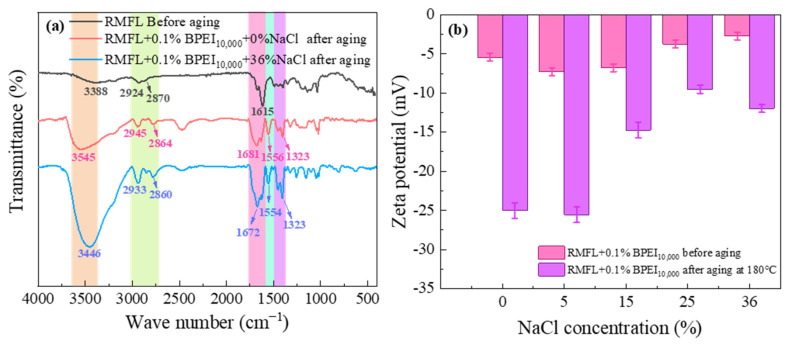
Characterization and analysis of the crosslinked polymer of RMFL and BPEI_10,000_. (**a**) Infrared spectrum of the crosslinking polymer; (**b**) zeta potential of the crosslinked polymer.

The infrared spectrum of the crosslinked polymer is shown in Figure 12a. The broad characteristic peaks at 3545 and 3446 cm^−1^ should originate from the stretching vibrations of hydroxyl, amine, and amide groups. After RMFL crosslinking with BPEI_10,000_, the crosslinked polymer contained abundant amine groups. Hydrogen bonding interactions between amine groups, carboxyl, and amide groups gave rise to the broad characteristic peaks observed [37]. The characteristic peaks at 2924, 2945, and 2933 cm^−1^ were related to the asymmetric stretching vibrations of the C-H bonds in the methylene groups. The characteristic peaks at 2870, 2864, and 2860 cm^−1^ came from the symmetric stretching vibrations of the C-H bonds in the methylene groups. The characteristic peak intensity and peak area associated with methylene stretching vibrations in the crosslinked product of RMFL and BPEI_10,000_ were greater, indicating an increased number of methylene groups. The characteristic peak at 1681 cm^−1^ corresponded with the stretching vibration of the C=O bond in the amide group, while the peaks at 1723 cm^−1^ and 1702 cm^−1^ likely stem from the stretching vibration of the C=O bond in the carboxyl group. The amide groups might have undergone prolonged high-temperature hydrolysis, forming carboxyl groups [38]. The peaks at 1554 cm^−1^ and 1323 cm^−1^ resulted from coupled vibrations of the N-H and C-N bonds in the amine groups. After interaction between RMFL and BPEI_10,000_, the intensities of these peaks for the N-H and C-N bonds significantly increased. The peak at 1040 cm^−1^ originated from the stretching vibration of the C-N bond. Its intensity in the crosslinked product was notably enhanced. The crosslinked polymer exhibited detectable C=O, -OH, C-H, C-N, and N-H bonds, suggesting a marked increase in carboxyl, imine, amide, and methylene groups. The results confirmed that BPEI_10,000_ and RMFL underwent crosslinking under 0% and 36% NaCl conditions [39]. The zeta potential of the crosslinked polymer was measured to analyze the charge properties of the RMFL/BPEI_10,000_ crosslinked polymer. As shown in Figure 12b, RMFL contained betaine-type zwitterionic structures with sulfonate groups that hydrate and carry a negative charge [40]. Due to the compressive effect of Na^+^ on the double electric layer of polymers, the zeta potential of RMFL gradually decreased. At different NaCl concentrations, the RMFL molecular chains carried negative charges before and after aging at 180 °C. It suggested that the crosslinked polymer carried a negative charge, primarily due to carboxyl groups formed by high-temperature hydrolysis of amide groups. The decrease in zeta potential indicated an increase in negative charge on the crosslinked polymer, which improved the dispersion stability of polymers, nanoparticles, and clay during drilling operations, thereby enhancing the colloidal stability of drilling fluids [41].

**Figure 13:**
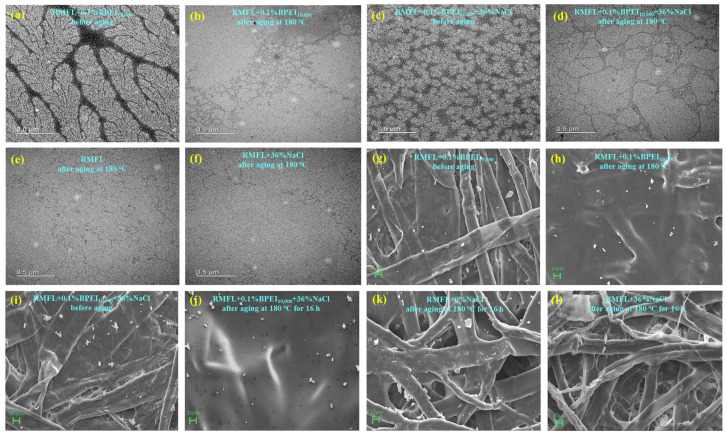
Analysis of polymer microtopography and filter cake microstructure. (**a**–**f**) Microstructure of RMFL and crosslinked polymer; (**g**–**l**) microstructure of filter cakes.

The microstructure of the polymers is shown in Figure 13a,d. Under 0% NaCl conditions, RMFL exhibited a willow-like morphology. Under 36% NaCl conditions, RMFL exhibited a densely crosslinked three-dimensional network structure. A three-dimensional network structure was observed in the crosslinked weak gel. As shown in Figure 13e,f, without the addition of BPEI_10,000_, RMFL underwent severe thermal degradation, with polymer chains visibly shortening and diminishing in size. The results demonstrated that RMFL was crosslinked with BPEI_10,000_ via a transamidation reaction at high temperatures, forming an extended crosslinking network and a weak gel. The crosslinking polymer could effectively enhance viscosity, increase shear strength, improve rheological properties, and reduce filtration loss in RMFL(BPEI_10,000_)/CFWBDFs under high-temperature, high-salinity conditions.

The microstructure of the filter cakes is shown in Figure 13g–l. Under 0% NaCl conditions, RMFL chains were tightly packed, forming a dense filter cake with fewer apparent pores or cracks. After adding BPEI_10,000_, the filter cake surface remained flat, smooth, and dense after aging at 180 °C. Under 36% NaCl conditions, the RMFL(BPEI_10,000_)/CFWBDFs formed compact filter cakes that were smooth and devoid of discernible dehydration pores or fissures before and after aging at 180 °C. In contrast, RMFL experienced severe thermal hydrolysis in the absence of BPEI_10,000_, resulting in filter cakes with larger micropores and higher filtration losses. The results indicated that RMFL could form a crosslinked weak gel with a three-dimensional network structure synergistic with BPEI_10,000_. The crosslinking polymer helped form a dense mud cake and effectively reduced filtration loss.

## 3. Conclusions

To address the challenge of insufficient temperature and salt resistance in clay-free rheology modifiers, thermal-induced crosslinking of RMFL and BPEI_10,000_ was achieved through a transamidation reaction, thereby maintaining the rheological properties and effectively reducing the filtration loss of the RMFL(BPEI_10,000_)/CFWBDFs at 180 °C and 36% NaCl. Here are the conclusions:

(1) Incorporating DMAA as the polymer backbone could endow rigidity and weak hydrophobic association properties to the polymers, and introducing DMAPS into the polymer side chains endowed the polymer with an anti-polyelectrolyte effect. The combination of DMAM and DMAPS could enhance the salt resistance of polymers.
(2) Even though excessive crosslinking resulted in gel formation at an AM mass fraction of 40%, it was approaching the expected outcome.
(3) The polymers without amide groups failed to crosslink with LPEI_1800_ under high-temperature and high-salinity conditions. Branched polyethyleneimine exhibited a superior crosslinking effect with RMFL compared to linear polyethyleneimine. BPEI_10,000_ demonstrated the most effective crosslinking performance. Under 180 °C and 36% NaCl conditions, when the BPEI_10,000_ concentration was 0.1% (M/V) and the RMFL concentration was 2%, the RMFL/BPEI_10,000_ system achieved the highest AV, PV, and YP and the lowest filtration loss.
(4) Under 180 °C and different NaCl conditions, the RMFL(BPEI_10,000_)/CFWBDFs exhibited appropriate shear thinning, viscoelasticity, and thixotropy properties. In particular, the viscosity of the RMFL(BPEI_10,000_)/CFWBDFs could be enhanced under high-temperature, high-pressure, and high-salinity conditions.
(5) A crosslinking polymer with a three-dimensional network could be formed by RMFL crosslinking with BPEI_10,000_, which improved the rheological properties of CFWBDFs.
(6) By synthesizing salt-tolerant polymers and selecting suitable crosslinking agents, the rheological properties of clay-free water-based drilling fluids can be regulated under high-temperature and high-salinity conditions.

Study limitations and recommended future work:

(1) The crosslinking between RMFL and BPEI_10,000_ in this study is not easy to control.
(2) The RMFL/BPEI_10,000_ system was not suitable for Ca^2+^- or Mg^2+^-based CFWBDFs, as these ions readily react with BPEI_10,000_.
(3) The proposed approach places high demands on drilling fluid formulation, requiring that the crosslinker and polymer exhibit high selectivity and do not react with other additives.
(4) Future research should focus not only on developing high-temperature and salt-resistant polymers but also on designing crosslinkers that are compatible with the polymers, exhibit controllable crosslinking, and have good compatibility with other drilling fluid additives. Although this approach is challenging, it remains an effective strategy to improve the overall performance of drilling fluids.
(5) Additionally, the lubricity, inhibition, contamination resistance, and HPHT filtration of drilling fluids should be studied to ensure their suitability for deep wells.

## 4. Materials and Methods

### 4.1. Materials

N,N-Dimethylacrylamide (DMAA, 99%), [2-(Methacryloyloxy)ethyl]dimethyl-(3-sulfonylpropyl)ammonium hydroxide (DMAPS, 97%), Acrylamide (AM, 99%), and 2,2′-Azobis(isobutyronitrile) dihydrochloride (V50, 97%) were procured from Shanghai Aladdin Biochemical Technology Co., Ltd. (Shanghai, China). Calcium chloride (CaCl_2_, 99%) and calcium bromide (CaBr_2_, 95%) were bought from Sinopharm Group (Shanghai, China). Sodium chloride (NaCl, 99%) was purchased from McLean Biochemical Co., Ltd. (Shanghai, China). Barite was bought from the BCL group (Guangxi, China). Linear polyethyleneimine (LPEI_x_, where x indicates the molecular weight of linear polyethyleneimine, x = 1800, 10,000, 70,000) and branched polyethyleneimine (BPEI_y_, where y indicates the molecular weight of branched polyethyleneimine, y = 1800, 10,000, 70,000) were obtained from Beijing Konojet Energy Environmental Protection Co., Ltd. (Beijing, China).

### 4.2. Methods

#### 4.2.1. Preparation of Polymers

Polyacrylamide (PAM) was synthesized using the following method: firstly, 30.0 g of AM was dissolved in 45.0 g of deionized water with stirring until complete dissolution. The solution was transferred to a three-neck flask and heated to 45 °C, and then, 0.020 g of V50 was added to the three-neck flask to initiate free radical polymerization. The reaction was carried out under nitrogen for 4 h, obtaining a transparent gel. The product was washed twice with anhydrous ethanol, dried at 80 °C for 24 h, and ground into powder to obtain PAM.

Poly(N,N-dimethylacrylamide) (PDMAA) was prepared with the following method: firstly, 30.0 g of DMAA was dissolved in 45.0 g of deionized water with stirring until complete dissolution. The solution was transferred to a three-neck flask and heated to 70 °C, and then, 0.020 g of V50 was added to the three-neck flask to initiate radical polymerization. The reaction was carried out under nitrogen for 4 h, and a transparent gel was obtained. The product was washed twice with anhydrous ethanol, dried at 80 °C for 24 h, and ground into powder to obtain PDMAA.

The zwitterionic modified poly(N,N-dimethylacrylamide) (PDMASs) was synthesized from DMAM and DMAPS. The total monomer mass was 30.0 g, with a monomer mass fraction of 40.0 wt%. The mass of DMAPS made up 0.5 wt%, 1.0 wt%, 1.5 wt%, and 2.0 wt% of the total monomer mass, which was 1.5 g, 3.0 g, 4.5 g, and 6.0 g of DMAPS being added in polymerization, respectively. Firstly, DMAM and DMAPS were dissolved in 45.0 g of deionized water with stirring until complete dissolution. The solution was transferred to a three-neck flask and heated to 70 °C, and then, 0.020 g of V50 was added to the three-neck flask to initiate free radical polymerization. The product was washed twice with anhydrous ethanol, dried at 80 °C for 24 h, and ground into powder to obtain PDMASs.

The amide groups were crosslinking sites between polymers and PEI. Based on the polymerization of PDMAS, the effect of the amide group mass fraction on the crosslinking of amide-based polymers and PEI was investigated by introducing amide groups. The mass fractions of AM were 0 wt%, 10 wt%, 20 wt%, 30 wt%, and 40 wt% of the total monomer mass, corresponding to additions of 0 g, 3.0 g, 6.0 g, 9.0 g, and 12.0 g, respectively. The total monomer mass was 30.0 g. Firstly, DMAM, DMAPS and AM were dissolved in 45.0 g of deionized water with stirring until complete dissolution. The solution was transferred to a three-neck flask and heated to 70 °C, and then, 0.020 g of V50 was added to the three-neck flask to initiate free radical polymerization. The product was washed twice with anhydrous ethanol, dried at 80 °C for 24 h, and ground into powder to obtain PDMASs. Based on the AM mass fraction, polymers were labeled as PDMASA-0, PDMASA-1, PDMASA-2, PDMASA-3, and PDMASA-4.

RMFL was synthesized with the following method: the monomer concentration was 50 wt%. DMAM accounted for 56.0 wt% of the total monomer mass, DMAPS for 4.0 wt%, and AM for 40.0 wt%. Firstly, 22.4 g of DMAM, 1.6 g of DMAPS, and 16.0 g of AM were added to a three-neck flask. The mixture was stirred at 400 rpm for 10 min until the monomers were fully dissolved. The mixture was heated to 55 °C, and 0.020 g of V50 was added to initiate radical polymerization. The reaction was carried out under nitrogen for 4 h, yielding a transparent gel. The gel was washed twice with anhydrous ethanol, dried at 80 °C for 24 h, and ground into powder to obtain the rheology modifier RMFL.

#### 4.2.2. Preparation of Drilling Fluids

At 25 °C, 8.0 g of polymer was dissolved in 400.0 mL of deionized water and stirred at 8000 rpm for 20 min to obtain the polymer-based clay-free water-based drilling fluids (polymer/CFWBDFs).

At 25 °C, 8.0 g of polymer was dissolved in 400.0 mL of deionized water and stirred at 8000 rpm for 20 min. Varying amounts of NaCl (0%, 5%, 15%, 25%, and 36%) were added, and the mixtures were stirred for 10 min to obtain the Polymer/NaCl CFWBDFs.

At 25 °C, a specified amount of polyethyleneimine was added to 5.0 g of deionized water with stirring to promote PEI dissolution and prepare a PEI solution. The prepared PEI solution was added to the polymer/CFWBDFs. The system was stirred for 5 min to prepare a composite solution, labeled as polymer(LPEI_x_)/CFWBDFs or polymer(BPEI_y_)/CFWBDFs.

To evaluate the crosslinking behavior of RMFL and BPEI_10,000_ at different densities and to investigate the applicability of the RMFL/BPEI_10,000_ composite system in high-density drilling fluids, 220.0 g and 300.0 g of barite (300 mesh) were added to 350.0 mL of RMFL (BPEI_10,000_)/CFWBDFs to adjust the drilling fluid densities to 1.40 g/cm^3^ and 1.60 g/cm^3^. In this system, the concentrations of RMFL, BPEI_10,000_, and NaCl were fixed at 2.0%, 0.1%, and 36%. Specifically, 480.0 g and 560.0 g of barite were added to adjust the drilling fluid density to 1.80 and 2.00 g/cm^3^, respectively.

At 25 °C, 140.0, 210.0, 280.0 and 350.0 g CaCl_2_ were added into 350.0 mL RMFL(BPEI_10,000_)/CFBWDFs, and the densities of RMFL(BPEI_10,000_)/CFBWDFs were adjusted to 1.26, 1.35, 1.41 and 1.43 g/cm^3^. At 25 °C, 150.0, 300.0, 450.0, and 660.0 g CaBr_2_ were added into 350.0 mL RMFL(BPEI_10,000_)/CFBWDFs, and the densities of RMFL(BPEI_10,000_)/CFBWDFs were adjusted to 1.28, 1.48, 1.63 and 1.75 g/cm^3^. The concentrations of RMFL, BPEI_10,000_ were fixed at 2.0%, 0.1%, and 36%.

#### 4.2.3. Characterization of Polymers

The IRTRacer-100 Fourier Transform Infrared Spectrometer (Shimadzu, Kyoto, Japan) was used to analyze the molecular structure of RMFL. The scanning range was from 460 cm^−1^ to 4000 cm^−1^, with a resolution of 4 cm^−1^.

RMFL was dissolved in D_2_O and scanned with the Bruker AV III nuclear magnetic resonance spectrometer (Bruker Corporation, Karlsruhe, Germany) to obtain the ^1^H NMR spectrum. The molecular weight of the polymer was determined using the 1260 Infinity II GPC/SEC gel permeation chromatograph (Agilent, Santa Clara, CA, USA). The chromatographic column was a hydrophilic gel-permeation column packed with a hydroxylated polymethacrylate resin, with a pore size of 8 μm. Firstly, 20 mg of RMFL was added to 10 mL of water (deionized water with 0.1 M NaNO_3_) and kept static at 25 °C for 24 h, stirring several times during the period to promote RMFL dissolution. Then, the solution was filtered using microfiltration membranes (0.22 μm) to remove insoluble polymer. Finally, the clarified solution was fed into the gel chromatography column for measurement.

The viscosity–shear rate relationship of RMFL/CFWBDFs was measured using a HAAKE rheometer (Thermo Scientific, Waltham, MA, USA) in continuous rotational scanning mode. Experiments were conducted at 25 °C with the shear rate ranging from 1 s^−1^ to 1050 s^−1^.

The thermal stability of RMFL in N_2_ was evaluated using a TGA-2 thermogravimetric analyzer. The temperature range was 30–550 °C, and the heating rate was 10 °C/min.

The transmittance of Polymer/CFWBDFs was measured at 600 nm using a UV-3600 Plus spectrophotometer (Shimadzu, Kyoto, Japan), and the microtopography of polymers was characterized.

#### 4.2.4. Evaluation of Clay-Free Water-Based Drilling Fluids

The rheological properties of CFWBDFs were evaluated by the HAAKE MARS 60 rheometer (Thermo Scientific, Waltham, MA, USA).

Apparent viscosity (AV, mPa·s), plastic viscosity (PV, mPa·s), and yield point (YP, Pa) are the essential rheological parameters for drilling fluids. Readings at θ600 and θ300 were obtained using a ZNN-D6 six-speed rotor viscometer (Qingdao Tongchun Petroleum Instrument Co., Ltd., Qingdao, China). AV, PV, and YP were calculated with the following equations. Each sample was tested three times on different instruments, and the results were averaged from the three measurements.

API filtration loss (FL_API_) was measured using an SD6A medium-pressure filter loss apparatus (Qingdao Tongchun Petroleum Instrument Co., Ltd., Qingdao, China) at 0.69 ± 0.03 MPa and 25 °C. Each sample was tested twice, and the results were averaged. Drilling fluids were placed in aging tanks, and they were transferred to a high-temperature roller oven. The drilling fluids were aging at high temperatures for 16 h. After aging, they were cooled to room temperature and stirred at 4500 rpm for 20 min, and the properties were measured.

The viscosity variation of RMFL (BPEI_10,000_)/CFWBDFs under high-temperature and high-salinity conditions was measured using a Chandler 5550 high-temperature and high-pressure rheometer (Chandler Engineering, Chandler, AZ, USA). Under time–temperature superposition conditions, the effect of the crosslinking reaction between RMFL and BPEI_10,000_ on the rheological behavior of RMFL (BPEI_10,000_)/CFWBDFs was investigated. The test temperature ranged from 30 to 150 °C, with a heating rate of 1.0 °C/min. The experiment was conducted for 120 min at 500 psi, with a shear rate of 170 s^−1^.

#### 4.2.5. Mechanism Analysis

The dried crosslinking polymers were ground into powder. The functional groups of the crosslinking polymers were characterized using an infrared spectrometer. The zeta potential of 2% RMFL solutions (with 0.1% BPEI_10,000_) was measured by using the Zetasizer Nano ZS-90 Zeta potential analyzer (Malvern, Malvern Worcestershire, England). The microstructure of RMFL and crosslinking polymers was observed using transmission electron microscopy. The filter cakes were dried at 60 °C, and the microtopography of the filter cakes was observed using an EVO 15 scanning electron microscope (Zeiss Oberkochen, Germany).

## Figures and Tables

**Table 1:** Summary of the current state of research and technologies.

Additive	Application	Raw Materials	Performance	Shortcomings
BioDrill FM400 [4]	Filtration reducer	BioDrill FM400 provided by Borregaard, Sarpsborg, Norway	Temperature resistant to 150 °C The PV, YP, and GS of the drilling fluid are significantly improved. Filtration loss and filter cake thickness are reduced by 63% and 42%, respectively.	Natural and natural-modified materials are easily degradable and have insufficient temperature resistance.
Tara gum [5]	Rheology modifier	Tara gum	Temperature resistant to 140 °C	
HMHEC [6]	Rheology modifier and filtration reducer	Cellulose and hydrophobic monomer	Temperature resistant to 150 °C EC50 was 60,000 mg/L, the BOD_5_/COD was 18.21%	
CMTS [4]	Rheology modifier	Native tapioca starch, monochloroacetic acid	Temperature resistant to 126 °C	
SBASC [9]	Filtration reducer	AM and ST	Resistant to temperatures up to 121 °C 25.0% NaCl and 5.7% KCl	Synthetic polymers have insufficient temperature resistance. Usually, below 180 °C, salt tolerance has not reached saturation.
SSZN [10]	Rheology modifier	AM, AMPS, DMDAAC, BA	Temperature resistant to 150 °C Salt tolerance up to 15%NaCl	
FPOD [11]	Rheology modifier	DMAA, DMDAAC, SBMA	Temperature resistant to 150 °C, CaBr_2_ to saturated	
SDKP [12]	Rheology modifier	AMPS, NVCL, DVB	Temperature resistance up to 160 °C and salt resistance up to 15% NaCl	

**Table 2:** Novelty and advantage of this study.

Additive	Application	Raw Materials	Performance	Novelty
RMFL and BPEI_10,000_	Rheology modifier	DMAA, SBMA, AM and BPEI_10,000_	Temperature resistant to 180 °C Salt tolerance up to 36% NaCl	1. RMFL had a betaine structure and exhibited an anti-polyelectrolyte effect. 2. RMFL contained crosslinking sites, which were capable of crosslinking with BPEI_10,000_ at high temperatures, preventing thermal degradation of RMFL. 3. The combination of RMFL and BPEI_10,000_ could effectively regulate the rheological properties of drilling fluid under high-temperature, high-salinity conditions.

**Table 3:** Fitting results of the Herschel–Bulkley model for RMFL/BPEI_10,000_ systems.

Rheology Model	Condition	NaCl Concentration (%)				
		0% NaCl	5% NaCl	15% NaCl	25% NaCl	36% NaCl
Herschel-Bulkley model $\tau =\tau_{y}+K\gamma^{n}$	Before aging	$\tau =2.809+0.3259\gamma^{0.6866}$ R^2^ = 0.9932	$\tau =2.798+0.3429\gamma^{0.6959}$ R^2^ = 0.9937	$\tau =2.961+0.3883\gamma^{0.6993}$ R^2^ = 0.9938	$\tau =2.738+0.3946\gamma^{0.7169}$ R^2^ = 0.9946	$\tau =2.596+0.4018\gamma^{0.7274}$ R^2^ = 0.9950
	After aging at 180 °C	$\tau =1.552+0.1863\gamma^{0.7567}$ R^2^ = 0.9996	$\tau =3.043+0.6449\gamma^{0.6792}$ R^2^ = 0.9976	$\tau =2.451+0.2857\gamma^{0.7825}$ R^2^ = 0.9955	$\tau =1.902+0.2237\gamma^{0.8067}$ R^2^ = 0.9995	$\tau =1.246+0.2793\gamma^{0.8124}$ R^2^ = 0.9965

## Data Availability

Data will be made available on request.
